# Some properties of the Catalan–Qi function related to the Catalan numbers

**DOI:** 10.1186/s40064-016-2793-1

**Published:** 2016-07-19

**Authors:** Feng Qi, Mansour Mahmoud, Xiao-Ting Shi, Fang-Fang Liu

**Affiliations:** Institute of Mathematics, Henan Polytechnic University, Jiaozuo City, 454010 Henan Province China; College of Mathematics, Inner Mongolia University for Nationalities, Tongliao City, 028043 Inner Mongolia Autonomous Region China; Department of Mathematics, Faculty of Science, Mansoura University, Mansoura, 35516 Egypt; Department of Mathematics, School of Science, Tianjin Polytechnic University, Tianjin City, 300387 China

**Keywords:** Property, Catalan number, Catalan function, Catalan–Qi function, Asymptotic expansion, Integral representation, Logarithmic convexity, Complete monotonicity, Logarithmically complete monotonicity, Minimality, Inequality, Ratio of gamma functions, Primary 33B15; Secondary 05A10, 05A15, 05A16, 05A19, 05A20, 11B83, 11Y55, 11Y60, 26A48, 26A51, 26D15, 33C05, 44A20

## Abstract

In the paper, the authors find some properties of the Catalan numbers, the Catalan function, and the Catalan–Qi function which is a generalization of the Catalan numbers. Concretely speaking, the authors present a new expression, asymptotic expansions, integral representations, logarithmic convexity, complete monotonicity, minimality, logarithmically complete monotonicity, a generating function, and inequalities of the Catalan numbers, the Catalan function, and the Catalan–Qi function. As by-products, an exponential expansion and a double inequality for the ratio of two gamma functions are derived.

## Background

It is stated in Koshy (2009), Stanley and Weisstein (2015) that the Catalan numbers $C_n$ for $n\ge 0$ form a sequence of natural numbers that occur in tree enumeration problems such as “In how many ways can a regular *n*-gon be divided into $n-2$ triangles if different orientations are counted separately?” whose solution is the Catalan number $C_{n-2}$. The Catalan numbers $C_n$ can be generated by

1 $$\begin{aligned} \frac{2}{1+\sqrt{1-4x}\,} &= \frac{1-\sqrt{1-4x}\,}{2x} =\sum _{n=0}^\infty C_nx^n \\ &= 1+ x+ 2x^2+ 5x^3+ 14x^4+ 42x^5+ 132x^6+ 429x^7+ 1430x^8+\cdots . \end{aligned}$$

Two of explicit formulas of $C_n$ for $n\ge 0$ read that

2 $$C_n=\frac{4^n\Gamma (n+1/2)}{\sqrt{\pi }\,\Gamma (n+2)} ={}_2F_1(1-n,-n;2;1),$$

where

$$\Gamma (z)=\int ^\infty _0t^{z-1} e^{-t}{{\text{d}}}t, \quad {\mathfrak {R}}(z)>0$$

is the classical Euler gamma function and

$${}_pF_q(a_1,\ldots ,a_p;b_1,\ldots ,b_q;z)=\sum _{n=0}^\infty \frac{(a_1)_n\cdots (a_p)_n}{(b_1)_n\cdots (b_q)_n}\frac{z^n}{n!}$$

is the generalized hypergeometric series defined for complex numbers $a_i\in {\mathbb {C}}$ and $b_i\in {\mathbb {C}}{\setminus} \{0,-1,-2,\ldots \}$, for positive integers $p,q\in {\mathbb {N}}$, and in terms of the rising factorials $(x)_n$ defined by

3 $$\begin{aligned} (x)_n=\prod _{\ell =0}^{n-1}(x+\ell ) = {\left\{ \begin{array}{ll} x(x+1)\cdots (x+n-1), &\quad n\ge 1\\ 1, &\quad n=0 \end{array}\right. } \end{aligned}$$

and

4 $$(-x)_n=(-1)^n(x-n+1)_n.$$

In Graham et al. (1994), Koshy (2009), Stanley and Weisstein (2015), Vardi (1991), it was mentioned that there exists an asymptotic expansion

5 $$C_x\sim \frac{4^x}{\sqrt{\pi }\,}\biggl (\frac{1}{x^{3/2}}-\frac{9}{8}\frac{1}{x^{5/2}} +\frac{145}{128}\frac{1}{x^{7/2}}+\cdots \biggr )$$

for the Catalan function $C_x$. What is the general expression for the asymptotic expansion (5)?

In Qi et al. (2015b, Remark 1) an analytical generalization of the Catalan numbers $C_n$ and the Catalan function $C_x$ was given by

6 $$C(a,b;z)=\frac{\Gamma (b)}{\Gamma (a)} \biggl (\frac{b}{a}\biggr )^z \frac{\Gamma (z+a)}{\Gamma (z+b)}, \quad {\mathfrak {R}}(a),{\mathfrak {R}}(b)>0, \quad {\mathfrak {R}}(z)\ge 0$$

and the integral representation

7 $$\begin{aligned} C(a,b;x) &= \frac{\Gamma (b)}{\Gamma (a)} \biggl (\frac{b}{a}\biggr )^x\frac{(x+a)^x}{(x+b)^{x+b-a}} \\&\quad\times \exp \biggl [b-a+\int _0^\infty \frac{1}{t}\biggl (\frac{1}{1-e^{-t}}-\frac{1}{t}-a\biggr ) \bigl (e^{-at}-e^{-bt}\bigr )e^{-xt}{{\text{d}}}t\biggr ] \end{aligned}$$

for $a,b>0$ and $x\ge 0$ was derived. For uniqueness and convenience of referring to the quantity (6), we call *C*(*a*, *b*; *x*) the Catalan–Qi function. It is clear that

$$C(a,b;0)=C(a,b;1)=1 \quad \text {and}\quad C(a,b;x)=\frac{1}{C(b,a;x)}.$$

The integral representation (7) generalizes an integral representation for $C\bigl (\frac{1}{2},2;x\bigr )$ in Shi et al. (2015). Currently we do not know and understand the combinatorial interpretations of *C*(*a*, *b*; *x*) and its integral representation (7). Here we would not like to discuss the combinatorial interpretations of them. What we concern here is the asymptotic expansion similar to (5) for *C*(*a*, *b*; *x*).

In Koshy (2009) and from https://en.wikipedia.org/wiki/Catalan_number, the integral representation

8 $$C_n=\frac{1}{2\pi }\int _0^4\sqrt{\frac{4-x}{x}}\,x^n{{\text{d}}}x$$

was listed. In Nkwanta and Tefera (2013, p. 10), there is an integral representation

$$C_n=\frac{2^{2n+5}}{\pi }\int _0^1\frac{x^2\bigl (1-x^2\bigr )^{2n}}{(1+x^2)^{2n+3}}{{\text{d}}}x.$$

In Qi et al. (2015c, Theorem 1.4), the integral representations

9 $$C_n=\frac{1}{\pi }\int _0^\infty \frac{\sqrt{t}\,}{(t+1/4)^{n+2}}{{\text{d}}}t =\frac{2}{\pi }\int _0^{\infty }\frac{t^2}{(t^2+1/4)^{n+2}}{{\mathrm{d}}}t, \quad n\ge 0$$

was established. In Qi (2015a, Theorem 1.3), the equivalence relation between (8) and (9) was verified. What is the integral representation of the Catalan–Qi function *C*(*a*, *b*; *x*) similar to either (8) or (9)?

From the power series (1), we observe that the Catalan numbers $C_n$ is an increasing sequence in $n\ge 0$ with $C_0=C_1$. What about the monotonicity and convexity of the Catalan numbers $C_n$, the Catalan function $C_x$, and the Catalan–Qi function *C*(*a*, *b*; *x*)? In Temme (1996, p. 67), it was listed that

$$\frac{\Gamma (z+a)}{\Gamma (z+b)}=\frac{1}{\Gamma (b-a)}\int _0^\infty \bigl (1-e^{-u}\bigr )^{b-a-1}e^{-(z+a)u}{{\text{d}}}u, \quad b>a\ge 0.$$

Accordingly, we obtain an alternative integral representation

10 $$C(a,b;x)=\frac{1}{B(a,b-a)}\biggl (\frac{b}{a}\biggr )^x \int _0^\infty \bigl (1-e^{-u}\bigr )^{b-a-1} e^{-(x+a)u}{{\text{d}}}u$$

for $b>a>0$ and $x\ge 0$, where *B*(*z*, *w*) denotes the classical beta function which can be defined (Abramowitz and Stegun 1972, p. 258, 6.2.1 and 6.2.2) by

11 $$B(z,w)=\int _0^1t^{z-1}(1-t)^{w-1}{{\text{d}}}t =\int _0^\infty \frac{t^{z-1}}{(1+t)^{z+w}}{{\mathrm{d}}}t$$

for ${\mathfrak {R}}(z)>0$ and ${\mathfrak {R}}(w)>0$ and satisfies

12 $$B(z,w)=\frac{\Gamma (z)\Gamma (w)}{\Gamma (z+w)}=B(w,z).$$

From the integral representations (8) and (9), one can not apparently see any message about the monotonicity and convexity of the Catalan–Qi function *C*(*a*, *b*; *x*) in $x\in [0,\infty )$.

As showed by (1), the Catalan numbers $C_n$ have a generating function $\frac{2}{1+\sqrt{1-4x}\,}$. What is the generating function of the Catalan–Qi numbers *C*(*a*, *b*; *n*)?

The aim of this paper is to supply answers to the above problems and others.

## A new expression of the Catalan numbers

In order to establish a new expression for the Catalan numbers $C_n$, we need the following lemma which was summarized up in the papers Qi (2015c, Section 2.2, p. 849), Qi (2016, p. 94), and Wei and Qi (2015, Lemma 2.1) from Bourbaki (2004, p. 40, Exercise 5).

### **Lemma 1**

*Letu*(*x*) *and*$v(x)\ne 0$*be differentiable functions, let*$U_{(n+1)\times 1}(x)$*be an*$(n+1)\times 1$*matrix whose elements*$u_{k,1}(x)=u^{(k-1)}(x)$*for*$1\le k\le n+1,$*let*$V_{(n+1)\times n}(x)$*be an*$(n+1)\times n$*matrix whose elements*

$$v_{i,j}(x)= {\left\{ \begin{array}{ll} \genfrac(){0.0pt}0{i-1}{j-1}v^{(i-j)}(x), &\quad i-j\ge 0\\ 0, &\quad i-j<0 \end{array}\right. }$$

*for*$1\le i\le n+1$*and*$1\le j\le n,$*and let*$|W_{(n+1)\times (n+1)}(x)|$*denote the determinant of the*$(n+1)\times (n+1)$*matrix*

$$W_{(n+1)\times (n+1)}(x)=\begin{bmatrix}U_{(n+1)\times 1}(x)&V_{(n+1)\times n}(x)\end{bmatrix}.$$

*Then the nth derivative of the ratio*$\frac{u(x)}{v(x)}$*can be computed by*

13 $$\frac{{{\text{d}}}^n}{{{\text{d}}}x^n}\biggl [\frac{u(x)}{v(x)}\biggr ] =(-1)^n\frac{\bigl |W_{(n+1)\times (n+1)}(x)\bigr |}{v^{n+1}(x)}.$$

Making use of the formula (13) in Lemma 1, we can obtain the following new expression for the Catalan numbers $C_n$.

### **Theorem 1**

*For*$n\in {\mathbb {N}}$, *the nth derivative of the generating function of the Catalan numbers*$C_n$*can be expressed as*

$$\frac{{{\text{d}}}^n}{{{\text{d}}}x^n}\biggl (\frac{1-\sqrt{1-4x}\,}{2x}\biggr ) =\frac{(-1)^{n+1}}{2{x^{n+1}}}\sum _{k=0}^{n}4^k \biggl \langle \frac{1}{2}\biggr \rangle _kx^{k}(1-4x)^{1/2-k}$$

*and the Catalan numbers*$C_n$*can be represented as*

$$C_n=\frac{4^n}{(n+1)!}\biggl (\frac{1}{2}\biggr )_n$$

*where*$\langle x\rangle _n$*is the falling factorial defined by*

$$\begin{aligned}\langle x\rangle _n= \prod _{k=0}^{n-1}(x-k)= {\left\{ \begin{array}{ll} x(x-1)\cdots (x-n+1), &\quad n\ge 1,\\ 1,&\quad n=0 \end{array}\right. } \end{aligned}$$

*and*$(x)_n$*is the rising factorial defined by* (3).

### *Proof*

Let $u(x)=1-\sqrt{1-4x}$ and $v(x)=x$. Since

$$u^{(k)}(x)=(-1)^{k+1}4^k\biggl \langle \frac{1}{2}\biggr \rangle _k(1-4x)^{1/2-k} \rightarrow (-1)^{k+1}4^k\biggl \langle \frac{1}{2}\biggr \rangle _k$$

for $k\in {\mathbb {N}}$ as $x\rightarrow 0$, making use of the formula (13) yields

$$\begin{aligned}&\frac{{{\text{d}}}^n}{{{\text{d}}}x^n}\biggl (\frac{1-\sqrt{1-4x}\,}{2x}\biggr )\\&\quad =\frac{(-1)^n}{2x^{n+1}} \begin{vmatrix} u(x)&\quad x&\quad 0&\quad 0&\quad \cdots&\quad 0&\quad 0&\quad 0\\ u'(x)&\quad \left( {\begin{array}{c}1\\ 0\end{array}}\right)&\quad x&\quad 0&\quad \cdots&\quad 0&\quad 0&\quad 0\\ u''(x)&\quad 0&\quad \left( {\begin{array}{c}2\\ 1\end{array}}\right)&\quad x&\quad \cdots&\quad 0&\quad 0&\quad 0\\ u'''(x)&\quad 0&\quad 0&\quad \left( {\begin{array}{c}3\\ 2\end{array}}\right)&\quad \cdots&\quad 0&\quad 0&\quad 0\\ \vdots&\quad \vdots&\quad \vdots&\quad \vdots&\quad \ddots&\quad \vdots&\quad \vdots&\quad \vdots \\ u^{(n-2)}(x)&\quad 0&\quad 0&\quad 0&\quad \cdots&\quad \left( {\begin{array}{c}n-2\\ n-3\end{array}}\right)&\quad x&\quad 0\\ u^{(n-1)}(x)&\quad 0&\quad 0&\quad 0&\quad \cdots&\quad 0&\quad \left( {\begin{array}{c}n-1\\ n-2\end{array}}\right)&\quad x\\ u^{(n)}(x)&\quad 0&\quad 0&\quad 0&\quad \cdots&\quad 0&\quad 0&\quad \left( {\begin{array}{c}n\\ n-1\end{array}}\right) \end{vmatrix} \\&\quad =\frac{(-1)^n}{2x^{n+1}} \begin{vmatrix} u(x)&\quad x&\quad 0&\quad 0&\quad \cdots&\quad 0&\quad 0&\quad 0\\ u'(x)&\quad 1&\quad x&\quad 0&\quad \cdots&\quad 0&\quad 0&\quad 0\\ u''(x)&\quad 0&\quad 2&\quad x&\quad \cdots&\quad 0&\quad 0&\quad 0\\ u'''(x)&\quad 0&\quad 0&\quad 3&\quad \cdots&\quad 0&\quad 0&\quad 0\\ \vdots&\quad \vdots&\quad \vdots&\quad \vdots&\quad \ddots&\quad \vdots&\quad \vdots&\quad \vdots \\ u^{(n-2)}(x)&\quad 0&\quad 0&\quad 0&\quad \cdots&\quad n-2&\quad x&\quad 0\\ u^{(n-1)}(x)&\quad 0&\quad 0&\quad 0&\quad \cdots&\quad 0&\quad n-1&\quad x\\ u^{(n)}(x)&\quad 0&\quad 0&\quad 0&\quad \cdots&\quad 0&\quad 0&\quad n \end{vmatrix} = \frac{(-1)^n}{2x^{n+1}} \\&\quad\qquad \times \begin{vmatrix} u(x)&\quad x&\quad 0&\quad 0&\quad \cdots&\quad 0&\quad 0&\quad 0\\ u'(x)-\frac{u(x)}{x}&\quad 0&\quad x&\quad 0&\quad \cdots&\quad 0&\quad 0&\quad 0\\ u''(x)-\frac{2}{x}\bigl [u'(x)-\frac{u(x)}{x}\bigr ]&\quad 0&\quad 0&\quad x&\quad \cdots&\quad 0&\quad 0&\quad 0\\ u'''(x)-\frac{3}{x}\bigl \{u''(x)-\frac{2}{x}\bigl [u'(x)-\frac{u(x)}{x}\bigr ]\bigr \}&\quad 0&\quad 0&\quad 0&\quad \cdots&\quad 0&\quad 0&\quad 0\\ \vdots&\quad \vdots&\quad \vdots&\quad \vdots&\quad \ddots&\quad \vdots&\quad \vdots&\quad \vdots \\ u^{(n-2)}(x)-\sum _{k=1}^{n-2}(-1)^k \frac{(n-2)!}{(n-k-2)!} \frac{u^{(n-k-2)}(x)}{x^k}&\quad 0&\quad 0&\quad 0&\quad \cdots&\quad 0&\quad x&\quad 0\\ u^{(n-1)}(x)-\sum _{k=1}^{n-1}(-1)^k \frac{(n-1)!}{(n-k-1)!} \frac{u^{(n-k-1)}(x)}{x^k}&\quad 0&\quad 0&\quad 0&\quad \cdots&\quad 0&\quad 0&\quad x\\ u^{(n)}(x)-\sum _{k=1}^{n}(-1)^k\frac{n!}{(n-k)!} \frac{u^{(n-k)}(x)}{x^k}&\quad 0&\quad 0&\quad 0&\quad \cdots&\quad 0&\quad 0&\quad 0 \end{vmatrix} \\&\quad = \frac{1}{2x^{n+1}}\Biggl [u^{(n)}(x)-\sum _{k=1}^{n}(-1)^k\frac{n!}{(n-k)!} \frac{u^{(n-k)}(x)}{x^k}\Biggr ] \begin{vmatrix} x&\quad 0&\quad 0&\quad \cdots&\quad 0&\quad 0&\quad 0\\ 0&\quad x&\quad 0&\quad \cdots&\quad 0&\quad 0&\quad 0\\ 0&\quad 0&\quad x&\quad \cdots&\quad 0&\quad 0&\quad 0\\ 0&\quad 0&\quad 0&\quad \cdots&\quad 0&\quad 0&\quad 0\\ \vdots&\quad \vdots&\quad \vdots&\quad \ddots&\quad \vdots&\quad \vdots&\quad \vdots \\ 0&\quad 0&\quad 0&\quad \cdots&\quad 0&\quad x&\quad 0\\ 0&\quad 0&\quad 0&\quad \cdots&\quad 0&\quad 0&\quad x \end{vmatrix} \end{aligned}$$

$$\begin{aligned}&\quad= \frac{1}{2x}\Biggl [u^{(n)}(x)-\sum _{k=1}^{n}(-1)^k\frac{n!}{(n-k)!} \frac{u^{(n-k)}(x)}{x^k}\Biggr ] \\&\quad =\frac{1}{2}\sum _{k=0}^{n}(-1)^k\frac{n!}{(n-k)!}\frac{u^{(n-k)}(x)}{x^{k+1}}\\&\quad =\frac{1}{2{x^{n+1}}}\sum _{k=0}^{n}(-1)^k\frac{n!}{(n-k)!}x^{n-k}u^{(n-k)}(x)\\&\quad =(-1)^n\frac{1}{2{x^{n+1}}}\sum _{k=0}^{n}(-1)^k\frac{n!}{k!}x^{k}u^{(k)}(x)\\&\quad \rightarrow \frac{(-1)^n}{2(n+1)!} \sum _{k=0}^{n}(-1)^k\frac{n!}{k!}\lim _{x\rightarrow 0}\bigl [x^{k}u^{(k)}(x)\bigr ]^{(n+1)}\\&\quad =\frac{(-1)^n}{2(n+1)!} \sum _{k=0}^{n}(-1)^k\frac{n!}{k!} \lim _{x\rightarrow 0} \sum _{\ell =0}^{n+1}\left( {\begin{array}{c}n+1\\ \ell \end{array}}\right) \bigl (x^{k}\bigr )^{(\ell )} u^{(n-\ell +k)}(x)\\&\quad =\frac{(-1)^n}{2(n+1)!} \sum _{k=0}^{n}(-1)^k\frac{n!}{k!}\left( {\begin{array}{c}n+1\\ k\end{array}}\right) k!u^{(n)}(0)\\&\quad =\frac{(-1)^n}{2(n+1)} \sum _{k=0}^{n}(-1)^k\left( {\begin{array}{c}n+1\\ k\end{array}}\right) u^{(n)}(0)\\&\quad =\frac{(-1)^n}{2(n+1)}(-1)^{n+1}4^n\biggl \langle \frac{1}{2}\biggr \rangle _n \sum _{k=0}^{n}(-1)^k\left( {\begin{array}{c}n+1\\ k\end{array}}\right) \\&\quad =-\frac{4^n}{2(n+1)}\biggl \langle \frac{1}{2}\biggr \rangle _n \sum _{k=0}^{n}(-1)^k\left( {\begin{array}{c}n+1\\ k\end{array}}\right) \\&\quad =(-1)^{n+1}\frac{4^n}{2(n+1)}\biggl \langle \frac{1}{2}\biggr \rangle _n\\&\quad =\frac{4^n}{n+1}\biggl \langle -\frac{1}{2}\biggr \rangle _n =\frac{4^n}{n+1}\biggl (\frac{1}{2}\biggr )_n \end{aligned}$$

as $x\rightarrow 0$. By virtue of the second function in the Eq. (1), we see that

$$\begin{aligned} C_n &= \frac{1}{n!}\lim _{x\rightarrow 0}\frac{{{\text{d}}}^n}{{{\text{d}}}x^n}\biggl (\frac{1-\sqrt{1-4x}\,}{2x}\biggr )\\ &= (-1)^{n}\frac{4^n}{(n+1)!}\biggl \langle -\frac{1}{2}\biggr \rangle _n =\frac{4^n}{(n+1)!}\biggl (\frac{1}{2}\biggr )_n. \end{aligned}$$

The proof of Theorem 1 is complete. $\square$

## Asymptotic expansions of the Catalan–Qi function $\varvec{C(a,b;x)}$

We first derive two asymptotic expansions of the Catalan–Qi function *C*(*a*, *b*; *x*). Consequently, from these two asymptotic expansions, we deduce a general expression for (5) and an asymptotic expansion of the ratio $\frac{\Gamma (a)}{\Gamma (b)}$ for $a,b>0$.

### **Theorem 2**

*Let*$B_k^{(\sigma )}(x)$*denote the generalized Bernoulli polynomials defined by*

14 $$e^{xz}\biggl (\frac{z}{e^z-1}\biggr )^\sigma =\sum _{k=0}^\infty \frac{B_k^{(\sigma )}(x)}{k!}z^k, \quad \sigma \in {\mathbb {C}}, \quad |z|<2\pi.$$

*For*$b>a>0$, *the Catalan–Qi functionC*(*a*, *b*; *x*) *has the asymptotic expansion*

15 $$C(a,b;x)\sim \frac{\Gamma (b)}{\Gamma (a)} \biggl (\frac{b}{a}\biggr )^x \sum _{k=0}^\infty (-1)^k\frac{B_k^{(a-b+1)}(a)}{k!}\frac{\Gamma (b-a+k)}{\Gamma (b-a)}\frac{1}{x^{k+b-a}}$$

*as*$x\rightarrow \infty$. *Consequently, the Catalan function*$C_x$*has the asymptotic expansion*

16 $$C_x=C\biggl (\frac{1}{2},2;x\biggr )\sim \frac{4^x}{\sqrt{\pi }\,} \sum _{k=0}^\infty (-1)^k\frac{B_k^{(-1/2)}(1/2)}{k!}\frac{\Gamma (k+3/2)}{\Gamma (3/2)}\frac{1}{x^{k+3/2}}$$

as $$x\rightarrow \infty$$.

### *Proof*

In Temme (1996, p. 67), it was listed that, under the condition ${\mathfrak {R}}(b-a)>0$,

$$\frac{\Gamma (z+a)}{\Gamma (z+b)}\sim z^{a-b}\sum _{k=0}^\infty (-1)^k\frac{B_k^{(a-b+1)}(a)}{k!}\frac{\Gamma (b-a+k)}{\Gamma (b-a)}\frac{1}{z^k}\quad \text {as } z\rightarrow \infty$$

in the sector $|\arg z|<\pi$, where the generalized Bernoulli polynomials $B_k^{(\sigma )}(x)$ are defined by (14) in Temme (1996, p. 4). Consequently, the function *C*(*a*, *b*; *x*) has the asymptotic expansion (15) under the condition $b>a>0$ as $x\rightarrow \infty$. In particular, when taking $a=\frac{1}{2}$ and $b=2$ in (15), we obtain the asymptotic expansion (16). Theorem 2 is thus proved. $\square$

### *Remark 1*

In Qi (2015a), there are another two asymptotic expansions for $C_n$ and $C_x$, which were established by virtue of the integral representations (8) and (7) for $a=\frac{1}{2}$ and $b=2$.

### *Remark 2*

The asymptotic expansion (16) is a general expression of the asymptotic expansion (5). Hence, the asymptotic expansion (15) is a generalization of (5).

### **Theorem 3**

*Let*$B_i$*denote the Bernoulli numbers defined by*

17 $$\frac{x}{e^x-1}=\sum _{i=0}^\infty B_i\frac{x^i}{i!} =1-\frac{x}{2}+\sum _{j=1}^\infty B_{2j}\frac{x^{2j}}{(2j)!}, \quad \vert x\vert <2\pi .$$

*Then the Catalan–Qi function C*(*a*, *b*; *x*) *has the exponential expansion*

18 $$\begin{aligned} C(a,b;x) &= \frac{\Gamma (b)}{\Gamma (a)} \biggl (\frac{b}{a}\biggr )^x\sqrt{\frac{x+b}{x+a}}\, [I(x+a,x+b)]^{a-b} \\&\quad\times \exp \Biggl [\sum _{j=1}^\infty \frac{B_{2j}}{2j(2j-1)} \biggl (\frac{1}{(x+a)^{2j-1}}-\frac{1}{(x+b)^{2j-1}}\biggr )\Biggr ], \quad a,b>0, \end{aligned}$$

*where*$I(\alpha ,\beta )$*denotes the exponential mean defined by*

19 $$I(\alpha ,\beta )=\frac{1}{e}\biggl (\frac{\beta ^\beta }{\alpha ^\alpha }\biggr )^{1/(\beta -\alpha )}$$

*for*$\alpha ,\beta >0$*with*$\alpha \ne \beta$. *Consequently, we have*

20 $$\frac{\Gamma (a)}{\Gamma (b)}=\sqrt{\frac{b}{a}}\, \frac{a^a}{b^b} \exp \Biggl [\sum _{j=1}^\infty \frac{B_{2j}}{2j(2j-1)} \biggl (\frac{1}{a^{2j-1}}-\frac{1}{b^{2j-1}}\biggr )\Biggr ], \quad a,b>0.$$

### Proof

Making use of (17) in the integral representation (7) yields

$$\begin{aligned} C(a,b;x)&=\frac{\Gamma (b)}{\Gamma (a)} \biggl (\frac{b}{a}\biggr )^x\frac{(x+a)^x}{(x+b)^{x+b-a}} \exp \biggl [b-a\\&\quad +\int _0^\infty \frac{1}{t}\biggl (\frac{1}{e^t-1}-\frac{1}{t}+1-a\biggr ) \bigl (e^{-at}-e^{-bt}\bigr )e^{-xt}{{\text{d}}}t\biggr ]\\&=\frac{\Gamma (b)}{\Gamma (a)} \biggl (\frac{b}{a}\biggr )^x\frac{(x+a)^x}{(x+b)^{x+b-a}} \exp \Biggl [b-a\\&\quad +\int _0^\infty \frac{1}{t}\Biggl (\frac{1}{2}-a+\sum _{j=1}^\infty B_{2j}\frac{t^{2j-1}}{(2j)!}\Biggr ) \bigl (e^{-at}-e^{-bt}\bigr )e^{-xt}{{\text{d}}}t\Biggr ]\\&=\frac{\Gamma (b)}{\Gamma (a)} \biggl (\frac{b}{a}\biggr )^x\frac{(x+a)^x}{(x+b)^{x+b-a}} \exp \Biggl [b-a+\biggl (\frac{1}{2}-a\biggr )\ln \frac{x+b}{x+a}\\&\quad +\sum _{j=1}^\infty \frac{B_{2j}}{2j(2j-1)} \biggl (\frac{1}{(x+a)^{2j-1}}-\frac{1}{(x+b)^{2j-1}}\biggr )\Biggr ]\\&=\frac{\Gamma (b)}{\Gamma (a)} \biggl (\frac{b}{a}\biggr )^x\frac{(x+a)^{x+a-1/2}}{(x+b)^{x+b-1/2}}e^{b-a}\\&\quad \times \exp \Biggl [\sum _{j=1}^\infty \frac{B_{2j}}{2j(2j-1)} \biggl (\frac{1}{(x+a)^{2j-1}}-\frac{1}{(x+b)^{2j-1}}\biggr )\Biggr ] \end{aligned}$$

which can be reformulated as the form (18).

The exponential expansion (20) follows from letting $x\rightarrow 0$ in (18) and rearranging. Theorem 3 is thus proved. $\square$

### *Remark 3*

When taking $a=\frac{1}{2}$ and $b=2$, the asymptotic expansion (18) reduces to one of conclusions in Qi (2015a, Theorem 1.2).

### *Remark 4*

For more information on the exponential mean $I(\alpha ,\beta )$ in (19), please refer to the monograph (Bullen 2003) and the papers (Guo and Qi 2009, 2011).

## Integral representations and complete monotonicity of the Catalan–Qi function $\varvec{C(a,b;x)}$

Motivated by the first integral representations (8) and (9), we guess out the following integral representations for the Catalan–Qi function *C*(*a*, *b*; *x*).

### **Theorem 4**

*For*$b>a>0$*and*$x\ge 0$, *the Catalan–Qi functionC*(*a*, *b*; *x*) *has integral representations*

21 $$C(a,b;x)=\biggl (\frac{a}{b}\biggr )^{b-1} \frac{1}{B(a,b-a)} \int _0^{b/a}\biggl (\frac{b}{a}-t\biggr )^{b-a-1}t^{x+a-1}{{\text{d}}}t$$

*and*

22 $$C(a,b;x)=\biggl (\frac{a}{b}\biggr )^{a}\frac{1}{B(a,b-a)}\int _0^\infty \frac{t^{b-a-1}}{(t+a/b)^{x+b}}{{\text{d}}}t.$$

### *Proof*

Straightforwardly computing and directly utilizing (11) and (12) acquire

$$\begin{aligned} \int _0^\infty \frac{t^{b-a-1}}{(t+a/b)^{x+b}} {{\text{d}}}t&=\biggl (\frac{b}{a}\biggr )^{x+b}\int _0^\infty \frac{t^{b-a-1}}{(1+bt/a)^{x+b}} {{\mathrm{d}}}t\\&=\biggl (\frac{b}{a}\biggr )^{x+b}\biggl (\frac{b}{a}\biggr )^{a-b} \int _0^\infty \frac{\bigl (\frac{bt}{a}\bigr )^{b-a-1}}{\bigl (1+\frac{bt}{a}\bigr )^{x+b}} {{\text{d}}}\biggl (\frac{bt}{a}\biggr )\\&=\biggl (\frac{b}{a}\biggr )^{x+a} \int _0^\infty \frac{u^{b-a-1}}{(1+u)^{b-a+(x+a)}} {{\text{d}}}u\\&=\biggl (\frac{b}{a}\biggr )^{x+a}B(b-a,x+a)\\&=\biggl (\frac{b}{a}\biggr )^{x+a}\frac{\Gamma (b-a)\Gamma (x+a)}{\Gamma (x+b)}. \end{aligned}$$

The integral representation (21) is thus proved.

Similar to the above argument, by virtue of (11) and (12), we obtain

$$\begin{aligned} \int _0^{b/a}\biggl (\frac{b}{a}-t\biggr )^{b-a-1}t^{x+a-1}{{\text{d}}}t &= \biggl (\frac{b}{a}\biggr )^{x+b-1}\int _0^1(1-s)^{b-a-1}s^{x+a-1}{{\text{d}}}s\\ &= \biggl (\frac{b}{a}\biggr )^{x+b-1}B(b-a,x+a) =\biggl (\frac{b}{a}\biggr )^{x+b-1}\frac{\Gamma (b-a)\Gamma (x+a)}{\Gamma (x+b)}. \end{aligned}$$

Hence, the integral representation (22) follows readily. The proof of Theorem 4 is thus complete. $\square$

### *Remark 5*

Letting $a=\frac{1}{2}$, $b=2$, and $x=n$ in (22) and (21) respectively reduce to the first integral representation in (9) and its equivalent form (8).

### *Remark 6*

In https://en.wikipedia.org/wiki/Catalan_number, it was said that the integral representation (8) means that the Catalan numbers $C_n$ are a solution of the Hausdorff moment problem on the interval [0, 4] instead of [0, 1]. Analogously, we guess that the integral representation (21) probably means that the Catalan–Qi numbers *C*(*a*, *b*; *n*) are a solution of the Hausdorff moment problem on the interval $\bigl [0,\frac{b}{a}\bigr ]$ instead of [0, 1] and [0, 4].

Recall from Mitrinović et al. (1993, Chapter XIII), Schilling et al. (2012, Chapter 1), and Widder (1941, Chapter IV) that an infinitely differentiable function *f* is said to be completely monotonic on an interval *I* if it satisfies $0\le (-1)^kf^{(k)}(x)<\infty$ on *I* for all $k\ge 0$. It is known (Widder 1941, p. 161, Theorem 12b) that a function *f* is completely monotonic on $(0,\infty )$ if and only if it is a Laplace transform $f(t)=\int _0^\infty e^{-ts}{{\text{d}}}\mu (s)$ of a positive measure $\mu$ defined on $[0,\infty )$ such that the above integral converges on $(0,\infty )$.

### **Theorem 5**

*For*$b>a>0$, *we have*

23 $$C(a,b;x) =\frac{1}{B(a,b-a)}\biggl (\frac{b}{a}\biggr )^{x} \sum _{k=0}^\infty (-1)^k\frac{\langle b-a-1\rangle _k}{k!} \frac{1}{x+a+k},$$

*where*

$$\begin{aligned} \langle x\rangle _n= \prod _{k=0}^{n-1}(x-k)= {\left\{ \begin{array}{ll} x(x-1)\cdots (x-n+1), &\quad n\ge 1 \\ 1,&\quad n=0 \end{array}\right. } \end{aligned}$$

*is the falling factorial. Consequently, the function*

24 $$(-1)^{\lfloor b-a\rfloor }\Biggl [\biggl (\frac{a}{b}\biggr )^{x} C(a,b;x)-\frac{1}{B(a,b-a)}\sum _{k=0}^N (-1)^k\frac{\langle b-a-1\rangle _k}{k!} \frac{1}{x+a+k}\Biggr ]$$

*for*$N\in \{0\}\cup {\mathbb {N}}$*and*$b>a>0$*is completely monotonic in*$x\in [0,\infty )$, *where*$\lfloor x\rfloor$*denotes the floor function whose value is the largest integer less than or equal to x*.

### *Proof*

The integral representation (21) can be rearranged as

25 $$\begin{aligned} C(a,b;x)&=\frac{1}{B(a,b-a)}\biggl (\frac{b}{a}\biggr )^{x-1} \int _0^{b/a}\biggl (1-\frac{a}{b}t\biggr )^{b-a-1}\biggl (\frac{a}{b}t\biggr )^{x+a-1}{{\text{d}}}t\\&=\frac{1}{B(a,b-a)}\biggl (\frac{b}{a}\biggr )^{x} \int _0^1(1-s)^{b-a-1}s^{x+a-1}{{\text{d}}}s. \end{aligned}$$

Further utilizing the well-known power series expansion

$$(1+x)^\alpha =\sum _{k=0}^\infty \langle \alpha \rangle _k\frac{x^k}{k!}, \quad |x|<1$$

arrives at

$$\begin{aligned} C(a,b;x)&=\frac{1}{B(a,b-a)}\biggl (\frac{b}{a}\biggr )^{x}\sum _{k=0}^\infty \frac{(-1)^k}{k!} \langle b-a-1\rangle _k\int _0^1s^{x+k+a-1}{{\text{d}}}s\\&=\frac{1}{B(a,b-a)}\biggl (\frac{b}{a}\biggr )^{x}\sum _{k=0}^\infty \frac{(-1)^k}{k!} \langle b-a-1\rangle _k\frac{1}{x+a+k} \end{aligned}$$

which can be reformulated as (23).

Rewriting (23) as

$$\begin{aligned}&\biggl (\frac{a}{b}\biggr )^{x}C(a,b;x)-\frac{1}{B(a,b-a)}\sum _{k=0}^N (-1)^k\frac{\langle b-a-1\rangle _k}{k!} \frac{1}{x+a+k}\\&\quad=\frac{1}{B(a,b-a)}\sum ^\infty _{k=N+1}(-1)^k\frac{\langle b-a-1\rangle _k}{k!} \frac{1}{x+a+k}\\&\quad=(-1)^{\lfloor b-a\rfloor }\frac{1}{B(a,b-a)}\sum ^\infty _{k=N+1}(-1)^{k-\lfloor b-a\rfloor } \frac{\langle b-a-1\rangle _k}{k!} \frac{1}{x+a+k}, \end{aligned}$$

considering the non-negativity of $(-1)^{k-\lfloor b-a\rfloor }\langle b-a-1\rangle _k$, and employing the complete monotonicity of $\frac{1}{x+a+k}$ in $x\in [0,\infty )$ reveal the complete monotonicity of the function (24). The proof of Theorem 5 is complete. $\square$

### *Remark 7*

When taking $a=\frac{1}{2}$ and $b=2$, Theorem 5 becomes a part of conclusions in Qi (2015a, Theorem 1.1).

## Logarithmically complete monotonicity of the Catalan–Qi function $\varvec{C(a,b;x)}$

An infinitely differentiable and positive function *f* is said to be logarithmically completely monotonic on an interval *I* if $0\le (-1)^k[\ln f(x)]^{(k)}<\infty$ hold on *I* for all $k\in {\mathbb {N}}$. The inclusions

26 $${\mathcal {L}}[I]\subset {\mathcal {C}}[I]\quad \text {and}\quad {\mathcal {S}}\setminus \{0\}\subset {\mathcal {L}}[(0,\infty )]$$

were discovered in Berg (2004), Guo and Qi (2010), Qi and Chen (2004), Qi and Guo (2004), where ${\mathcal {L}[I]}$, ${\mathcal {C}[I]}$, and ${\mathcal {S}}$ denote respectively the set of all logarithmically completely monotonic functions on an interval *I*, the set of all completely monotonic functions on *I*, and the set of all Stieltjes transforms. See also the monograph Schilling et al. (2012) and plenty of references therein.

Recall from monographs Mitrinović et al. (1993, pp. 372–373) and Widder (1941, p. 108, Definition 4) that a sequence $\{\mu _n\}_{0\le n\le \infty }$ is said to be completely monotonic if its elements are non-negative and its successive differences are alternatively non-negative, that is,

$$(-1)^k\Delta ^k\mu _n\ge 0$$

for $$n,k\ge 0$$, where

$$\Delta ^k\mu _n=\sum _{m=0}^k(-1)^m\left( {\begin{array}{c}k\\ m\end{array}}\right) \mu _{n+k-m}.$$

Recall from Widder (1941, p. 163, Definition 14a) that a completely monotonic sequence $\{a_n\}_{n\ge 0}$ is minimal if it ceases to be completely monotonic when $a_0$ is decreased.

### **Theorem 6**

*The function*

$$\begin{aligned} {\mathcal {C}}^{\pm 1}(a,b;x)= \left\{ \begin{array}{ll} 1, &\quad x=0 \\ {[}C(a,b;x)]^{\pm 1/x}, &\quad x>0 \\ \end{array}\right. \end{aligned}$$

*is logarithmically completely monotonic on*$(0,\infty )$*if and only if*$a\gtrless b$. *Consequently, the sequence*

27 $$\begin{aligned} {\mathcal {C}}_n= {\left\{ \begin{array}{ll} 1, &\quad n=0\\ \dfrac{1}{\root n \of {C_n}\,}, &\quad n\in {\mathbb {N}} \end{array}\right. } \end{aligned}$$

*is completely monotonic, minimal, and logarithmically convex.*

### *Proof*

In Qi and Li (2015, Theorem 1.1), it was proved that, when $a\gtrless b$, the function

28 $$\begin{aligned}{}[h_{a,b;c}(x)]^{\pm 1}= {\left\{ \begin{array}{ll} 1, &\quad x=0 \\ \biggl [c\dfrac{\Gamma (x+a)}{\Gamma (x+b)}\biggr ]^{\pm 1/x}, &\quad x>0 \end{array}\right. } \end{aligned}$$

for $c>0$ is logarithmically completely monotonic on $[0,\infty )$ if and only if $c\gtreqless \frac{\Gamma (b)}{\Gamma (a)}$. It is easy to see that

$${\mathcal {C}}^{\pm 1}(a,b;x) =\biggl (\frac{b}{a}\biggr )^{\pm 1}\bigl [h_{a,b;\Gamma (b)/\Gamma (a)}(x)\bigr ]^{\pm 1}.$$

Therefore, the function ${\mathcal {C}}^{\pm 1}(a,b;x)$ is logarithmically completely monotonic on $[0,\infty )$ if and only if $a\gtrless b$. Consequently, the function ${\mathcal {C}}^{-1}\bigl (\frac{1}{2},2;x\bigr )$ is logarithmically completely monotonic, and then completely monotonic and logarithmically convex, on $[0,\infty )$. As a result, the complete monotonicity, minimality, and logarithmic convexity of the sequence (27) follows immediately from Widder (1941, p. 164, Theorem 14b) which reads that a necessary and sufficient condition that there should exist a completely monotonic function *f*(*x*) in $0\le x<\infty$ such that $f(n)=a_n$ for $n\ge 0$ is that $\{a_n\}_0^\infty$ should be a minimal completely monotonic sequence. The proof of Theorem 6 is complete. $\square$

### *Remark 8*

It is interesting that, since the function $h_{a,b;c}(x)$ defined by (28) originates from the coding gain (see Lee and Tepedelenlioğlu 2011; Qi and Li 2015), Theorem 6 and its proof imply some connections and relations among the Catalan numbers, the coding gain, and the ratio of two gamma functions.

### **Theorem 7**

*Let*$a,b>0$*and*$x\ge 0$. *Then*

1. *when*$b>a$, *the function C*(*a*, *b*; *x*) *is decreasing in*$x\in [0,x_0)$, *increasing in*$x\in (x_0,\infty )$,*and logarithmically convex in*$x\in [0,\infty )$;
2. *when*$b<a$, *the function C*(*a*, *b*; *x*) *is increasing in*$x\in [0,x_0)$, *decreasing in*$x\in (x_0,\infty )$, *and logarithmically concave in*$x\in [0,\infty )$;

*where*$x_0$*is the unique zero of the equation*

29 $$\frac{\psi (x+b)-\psi (x+a)}{\ln b-\ln a}=1$$

*and satisfies*$x_0\in \bigl (0,\frac{1}{2}\bigr )$. *Consequently, the Catalan numbers*$C_n$*for*$n\in {\mathbb {N}}$*is strictly increasing and logarithmically convex*.

### *Proof*

In Guo and Qi (2010, Theorem 1) closely-related references therein, it was proved that the function

$$\theta _\alpha (x)=x^\alpha [\ln x-\psi (x)]$$

is completely monotonic on $(0,\infty )$ if and only if $\alpha \le 1$. This means that

$$\ln a-\psi (a)\lessgtr \ln b-\psi (b), \quad a\gtrless b,$$

that is,

30 $$\frac{\psi (b)-\psi (a)}{\ln b-\ln a} >1, \quad a\ne b.$$

This can also be verified by virtue of the inequality

$$\psi '(x)>\frac{1}{x}+\frac{1}{2x^2}>\frac{1}{x}, \quad x>0,$$

which is a special case of Guo and Qi (2010, Lemma 3), and by virtue of the equality

$$\frac{\psi (b)-\psi (a)}{\ln b-\ln a}=\frac{\int _a^b\psi '(x){{\text{d}}}x}{\int _a^b1/x{{\text{d}}}x}.$$

Since the function $\psi (x+b)-\psi (x+a)$ is increasing (or decreasing, respectively) if and only if $b<a$ (or $b>a$, respectively) and

$$\lim _{x\rightarrow \infty }[\psi (x+b)-\psi (x+a)]=0$$

for all $a,b>0$, we obtain that for all $a,b>0$ with $a\ne b$ the function $\frac{\psi (x+b)-\psi (x+a)}{\ln b-\ln a}$ is strictly decreasing on $[0,\infty )$ and

31 $$\lim _{x\rightarrow \infty }\frac{\psi (x+b)-\psi (x+a)}{\ln b-\ln a}=0.$$

It is clear that the first derivative

$$\frac{\partial [\ln C(a,b;x)]}{\partial x}=(\ln b-\ln a)-[\psi (x+b)-\psi (x+a)]\lesseqgtr 0$$

if and only if

$$\ln b-\ln a\lesseqgtr \psi (x+b)-\psi (x+a)$$

which can be rewritten as

$$\frac{\psi (x+b)-\psi (x+a)}{\ln b-\ln a}\gtreqless 1, \quad b>a$$

and

$$\frac{\psi (x+b)-\psi (x+a)}{\ln b-\ln a}\lesseqgtr 1, \quad b<a.$$

As a result, considering (30) and (31), we see that the Catalan–Qi function *C*(*a*, *b*; *x*) for all $a,b>0$ with $a\ne b$ is not monotonic on $[0,\infty )$ and that

1. when $b>a$, the function *C*(*a*, *b*; *x*) is decreasing in $x\in (0,x_0)$ and increasing in $x\in (x_0,\infty )$;
2. when $b<a$, the function *C*(*a*, *b*; *x*) is increasing in $x\in (0,x_0)$ and decreasing in $x\in (x_0,\infty )$;

where $x_0$ is the unique zero of the Eq. (29).

The Eq. (29) can be rearranged as

$$\psi (x+b)-\psi (x+a)=\ln b-\ln a.$$

Regarding *b* as a variable and differentiating with respect to *b* give

$$\psi '(x+b)=\frac{1}{b}=\frac{1}{(x+b)-x}$$

which can be reformulated as

$$x=(x+b)-\frac{1}{\psi '(x+b)}\triangleq u-\frac{1}{\psi '(u)},$$

where $$\lim _{u\rightarrow 0^+}\bigl [u-\frac{1}{\psi '(u)}\bigr ]=0$$ and

$$\frac{{{\text{d}}}}{{{\text{d}}}u}\biggl [u-\frac{1}{\psi '(u)}\biggr ] =1+\frac{\psi ''(x)}{[\psi '(x)]^2} =\frac{[\psi '(x)]^2+\psi ''(x)}{[\psi '(x)]^2}.$$

Employing the asymptotic expansion

$$\psi '(x) =\frac{1}{x}+\frac{1}{2x^{2}}+\sum _{m=1}^\infty \frac{B_{2m}}{x^{2m+1}}$$

in Abramowitz and Stegun (1972, p. 260, 6.4.11) yields

$$u-\frac{1}{\psi '(u)}=\frac{\frac{1}{2u}+\sum _{m=1}^\infty \frac{B_{2m}}{u^{2m}}}{\frac{1}{u}+\frac{1}{2u^{2}}+\sum _{m=1}^\infty \frac{B_{2m}}{u^{2m+1}}} \rightarrow \frac{1}{2}, \quad u\rightarrow \infty .$$

Due to $[\psi '(x)]^2+\psi ''(x)>0$ on $(0,\infty )$, see Alzer (2004), Qi (2015b), Qi and Li (2015), Qi et al. (2013) and plenty of closely-related references therein, the function $u-\frac{1}{\psi '(u)}$ is strictly increasing, and so

$$0<u-\frac{1}{\psi '(u)}<\frac{1}{2},$$

on $(0,\infty )$. Accordingly, the unique zero $x_0$ of the Eq. (29) belongs to $\bigl (0,\frac{1}{2}\bigr )$.

It is immediate that

$$\frac{\partial ^2[\ln C(a,b;x)]}{\partial x^2}=\psi '(x+a)-\psi '(x+b).$$

Since the tri-gamma function $\psi '(x)$ is completely monotonic on $(0,\infty )$, inequalities

$$(-1)^{k+1}\frac{\partial ^{k+1}[\ln C(a,b;x)]}{\partial x^{k+1}} =\psi ^{(k)}(x+a)-\psi ^{(k)}(x+b)\lessgtr 0$$

for $k\in {\mathbb {N}}$ hold if and only if $b\lessgtr a$. The proof of Theorem 7 is complete. $\square$

### *Remark 9*

From Theorem 7, we can derive that, for $b>a>0$,

$$\frac{\Gamma (x+a)}{\Gamma (x+b)}\lessgtr \frac{\Gamma (a)}{\Gamma (b)}\biggl (\frac{a}{b}\biggr )^x, \quad 0<x\lessgtr 1.$$

In other words,

$$0<C(a,b;x)\lessgtr 1, \quad 0<x\lessgtr 1, \quad b>a>0.$$

### **Theorem 8**

*For*$b>a>0$, *the function*

32 $$\biggl (\frac{a}{b}\biggr )^{x}C(a,b;x)$$

*is logarithmically completely monotonic on*$[0,\infty )$.

### *Proof*

By (6), it follows that

$$\biggl (\frac{a}{b}\biggr )^{x}C(a,b;x) =\frac{\Gamma (b)}{\Gamma (a)} \frac{\Gamma (x+a)}{\Gamma (x+b)}$$

which can be straightforwardly verified to be a logarithmically completely monotonic function on $[0,\infty )$. By the first inclusion in (26), we obtain the required complete monotonicity of the function (32). $\square$

### *Remark 10*

The integral representation (22) can be rewritten as

$$C(a,b;x)=\frac{1}{B(a,b-a)} \biggl (\frac{b}{a}\biggr )^{x+b-a} \int _0^\infty \frac{t^{b-a-1}}{(bt/a+1)^{x+b}}{{\text{d}}}t$$

for $b>a>0$ and $x\ge 0$. This formula and both of the integral representations (10) and (25) all mean that the function (32) for $b>a>0$ is completely monotonic on $[0,\infty )$. This conclusion is weaker than Theorem 8.

### **Theorem 9**

*For*$b>a>0$, *the function*

$$\biggl (\frac{a}{b}\biggr )^x\frac{(x+b)^{x+b-a}}{(x+a)^x}C(a,b;x)$$

*is logarithmically completely monotonic on*$[0,\infty )$.

### *Proof*

This follows from the integral representation (7). $\square$

### *Remark 11*

Theorems 8 and 9 imply that the sequences

$$\biggl \{\frac{C_n}{4^n}\biggr \}_{n\ge 0} \quad \text {and}\quad \biggl \{\frac{(n+2)^{n+3/2}}{(n+1/2)^n}\frac{C_n}{4^n}\biggr \}_{n\ge 0}$$

are logarithmically completely monotonic and minimal, which have been concluded in Qi (2015a, Theorems 1.1 and 1.2).

## A generating function of the Catalan–Qi sequence $\varvec{C(a,b;n)}$

In this section, we discover that ${}_2F_1\bigl (a,1;b;\frac{bt}{a}\bigr )$ is a generating function of the Catalan–Qi numbers *C*(*a*, *b*; *n*).

### **Theorem 10**

*For*$a,b>0$*and*$n\ge 0$, *the Catalan–Qi numbersC*(*a*, *b*; *n*) *can be generated by*

33 $${}_2F_1\biggl (a,1;b;\frac{bt}{a}\biggr )=\sum _{n=0}^\infty C(a,b;n)t^n$$

*and, conversely, satisfy*

34 $$C(a,b;n)=(-1)^n\sum _{k=0}^n(-1)^k\left( {\begin{array}{c}n\\ k\end{array}}\right) {}_2F_1\biggl (a,-k;b;-\frac{b}{a}\biggr ).$$

### *Proof*

Using the relation $(z)_n \Gamma (z)=\Gamma (z+n)$ for $n \ge 0$, we have

$$C(a,b;n)=\left( \frac{b}{a} \right) ^n \frac{(a)_n}{(b)_n},\quad a,b>0, \quad n \ge 0.$$

As a result, we obtain

$$\sum _{n=0}^{\infty } C(a,b;n)t^n=\sum _{n=0}^{\infty } \frac{(a)_n(1)_n}{(b)_n} \frac{(b t/a)^n}{n!} ={}_2F_1\biggl (a,1;b;\frac{bt}{a}\biggr ), \quad a,b>0.$$

Using the relation $(-n)_{n+i}=0$ for $i\in {\mathbb {N}}$, which can be derived from (4), we obtain

$${}_2F_1\biggl (a,-n;b;-\frac{b}{a}\biggr )=\sum _{r=0}^{n} \frac{(-1)^r (-n)_r }{r!}C(a,b;r).$$

Further using the relation

$$(-1)^r(-n)_r=(n-r+1)_r=\frac{\Gamma (n+1)}{\Gamma (n-r+1)}=\frac{n!}{(n-r)!},$$

we acquire

35 $${}_2F_1\biggl (a,-n;b;-\frac{b}{a}\biggr )=\sum _{r=0}^{n}\left( {\begin{array}{c}n\\ r\end{array}}\right) C(a,b;r).$$

The formula (Graham et al. 1994, p. 192, (5.48)) reads that

$$g(k)=\sum _\ell \left( {\begin{array}{c}k\\ \ell \end{array}}\right) (-1)^\ell f(\ell ) \quad \text {if and only if}\quad f(k)=\sum _\ell \left( {\begin{array}{c}k\\ \ell \end{array}}\right) (-1)^\ell g(\ell ).$$

Hence, the inversion of the relation (35) gives us the relation (34). The proof of Theorem 10 is complete. $\square$

### *Remark 12*

(An alternative proof of (33) for $b>1$) In Abramowitz and Stegun (1972, p. 558, 15.3.1), it is collected that

$${}_2F_1(a,b;c;z)=\frac{1}{B(b,c-b)}\int _0^1\frac{t^{b-1}(1-t)^{c-b-1}}{(1-tz)^a}{{\text{d}}}t, \quad {\mathfrak {R}}(c)>{\mathfrak {R}}(b)>0.$$

In order to prove the Eq. (33), it is sufficient to show

$$\lim _{t\rightarrow 0}\frac{{{\text{d}}}^n}{{{\text{d}}}t^n}\biggl [{}_2F_1\biggl (a,1;b;\frac{bt}{a}\biggr )\biggr ]=n!C(a,b;n).$$

In fact, a straightforward calculation reveals

$$\begin{aligned}&\lim _{z\rightarrow 0}\frac{{{\text{d}}}^n}{{{\text{d}}}z^n} \biggl [\frac{1}{B(1,b-1)}\int _0^1\frac{(1-t)^{b-2}}{(1-btz/a)^a}{{\text{d}}}t\biggr ]\\&\quad =(b-1)a^a\lim _{z\rightarrow 0}\frac{{{\text{d}}}^n}{{{\text{d}}}z^n} \int _0^1\frac{(1-t)^{b-2}}{(a-btz)^a}{{\text{d}}}t\\&\quad =(b-1)a^ab^n\frac{\Gamma (n+a)}{\Gamma (a)} \lim _{z\rightarrow 0}\int _0^1\frac{t^n(1-t)^{b-2}}{(a-btz)^{a+n}}{{\text{d}}}t\\&\quad =\frac{(b-1)a^ab^n}{a^{a+n}} \frac{\Gamma (n+a)}{\Gamma (a)}\int _0^1t^n(1-t)^{b-2}{{\text{d}}}t\\&\quad =\frac{(b-1)b^n}{a^{n}}\frac{\Gamma (n+a)}{\Gamma (a)} \frac{\Gamma (b-1) \Gamma (n+1)}{\Gamma (n+b)}\\&\quad =n!\frac{b^n}{a^{n}}\frac{\Gamma (n+a)}{\Gamma (a)}\frac{\Gamma (b)}{\Gamma (n+b)}\\&\quad =n!C(a,b;n) \end{aligned}$$

for $b>1$. This gives an alternative proof of (33) for $b>1$.

### *Remark 13*

Combining (2) and (34) brings out

$$\begin{aligned} {}_2F_1(1-n,-n;2;1)=(-1)^n\sum _{k=0}^n(-1)^k\left( {\begin{array}{c}n\\ k\end{array}}\right) {}_2F_1\biggl (\frac{1}{2},-k;2;-4\biggr ). \end{aligned}$$

## A double inequality of the Catalan–Qi function $\varvec{C(a,b;x)}$

Finally we present a double inequality of the Catalan–Qi function *C*(*a*, *b*; *x*).

### **Theorem 11**

*Let*$B_i$*for*$i\in {\mathbb {N}}$*be the Bernoulli numbers defined by* (17) *and letIbe the exponential mean defined by* (19). *Then the Catalan–Qi function C*(*a*, *b*; *x*) *satisfies the double inequality*

36 $$\begin{aligned}&\exp \Biggl [\sum _{j=1}^{2m}\frac{B_{2j}}{2j(2j-1)} \biggl (\frac{1}{(x+a)^{2j-1}}-\frac{1}{(x+b)^{2j-1}}\biggr )\Biggr ] \\&\quad<\frac{\Gamma (a)}{\Gamma (b)} \biggl (\frac{a}{b}\biggr )^x\sqrt{\frac{x+a}{x+b}}\, \frac{C(a,b;x)}{[I(x+a,x+b)]^{a-b}} \\&\quad <\exp \Biggl [\sum _{j=1}^{2m-1}\frac{B_{2j}}{2j(2j-1)} \biggl (\frac{1}{(x+a)^{2j-1}}-\frac{1}{(x+b)^{2j-1}}\biggr )\Biggr ]. \end{aligned}$$

Consequently, we have

37 $$\begin{aligned}&\sqrt{\frac{b}{a}}\, [I(a,b)]^{a-b}\exp \Biggl [\sum _{j=1}^{2m}\frac{B_{2j}}{2j(2j-1)} \biggl (\frac{1}{a^{2j-1}}-\frac{1}{b^{2j-1}}\biggr )\Biggr ]<\frac{\Gamma (a)}{\Gamma (b)} \\&\quad <\sqrt{\frac{b}{a}}\, [I(a,b)]^{a-b}\exp \Biggl [\sum _{j=1}^{2m-1}\frac{B_{2j}}{2j(2j-1)} \biggl (\frac{1}{a^{2j-1}}-\frac{1}{b^{2j-1}}\biggr )\Biggr ]. \end{aligned}$$

### *Proof*

In Koumandos (2006, Theorem 3), it was obtained that

$$1-\frac{x}{2}+\sum _{j=1}^{2m}\frac{B_{2j}}{(2j)!}x^{2j}<\frac{x}{e^x-1} <1-\frac{x}{2}+\sum _{j=1}^{2m-1}\frac{B_{2j}}{(2j)!}x^{2j}$$

for $m\in {\mathbb {N}}$ and $x>0$. Substituting this double inequality into the integral representation (7) and straightforward computing lead to the double inequality (36).

The double inequality (37) follows from letting $x\rightarrow 0$ in (36) and simplifying. The proof of Theorem 11 is complete. $\square$

### *Remark 14*

The double inequality (36) generalizes a double inequality in Qi (2015a, Theorem 1.2).

## Conclusions

The main conclusions of this paper are stated in Theorems 1, 2, 3, 4, 5, 6, 7, 8, 9, 10, and 11. Concretely speaking, a new expression, several asymptotic expansions, several integral representations, logarithmic convexity, complete monotonicity, minimality, logarithmically complete monotonicity, a generating function, and several inequalities of the Catalan numbers, the Catalan function, and the Catalan–Qi function are presented and an exponential expansion and a double inequality for the ratio of two gamma functions are derived. These conclusions generalize and extend some known results. More importantly, these conclusions provide new viewpoints of understanding and supply new methods of investigating the Catalan numbers in combinatorics and number theory. Moreover, these conclusions connect the Catalan numbers with the ratios of two gamma functions in the theory of special functions. In other words, the main conclusions in this paper will deepen and promote the study of the Catalan numbers and related concepts in combinatorics and number theory.

### *Remark 15*

This paper is a companion of the articles Liu et al. (2015), Mahmoud and Qi (2016), Qi (2015a, d, e), Qi and Guo (2016a, b), Qi et al. (2015b, c, d, e), Shi et al. (2015) and a revised version of the preprint Qi et al. (2015a).
